# Examining professional boundaries between nurses and physicians in neonatal intensive care units

**DOI:** 10.1186/2045-4015-3-43

**Published:** 2014-12-18

**Authors:** Orly Toren, Nurit Nirel, Yehuda Tsur, Michal Lipschuetz, Asaf Toker

**Affiliations:** Hadassah Medical Center, Jerusalem, Israel; Smokler Center for Health Policy Research, Myers-JDC-Brookdale Institute, Jerusalem, Israel; Department of Health Systems Management, Faculty of Health Sciences, Ben Gurion University of the Negev, Be’er Sheva, Israel

**Keywords:** Neonatal-nurse-specialist, Neonatal Intensive Care Units (NICUs), RNs, Authority, Responsibility, Professional boundaries

## Abstract

**Background:**

Medical and technological developments, financial constraints and a shortage of physicians have made it necessary to re-examine professional boundaries between physicians and nurses. Israel’s manpower shortage in Neonatal Intensive Care Units (NICUs) has changed the responsibility and authority of nurses. However, these changes have not evolved into a uniform policy defining the division of responsibility between physicians and nurses. This study was designed to examine the work processes and actual division of labor between NICU physicians and nurses; the attitude of physicians and nurses to greater empowerment of the nursing role; and to suggest a model to regulate work processes and develop the role of neonatal nurse specialists in NICUs.

**Methods:**

Open interviews with NICU physician-directors and head nurses and a cross-sectional survey of some 50% of the physicians and nurses at 22 hospital NICUs (N = 430).

**Results:**

*Main problems of NICUs*: physician shortage, deficient infrastructures, fragmented work processes. Nurses do not perform many practices allowed to them due to the need for organizational approval and their own unawareness. Conversely, they sometimes conduct procedures and make decisions outside of their authority.

Most physicians agree that nurse graduates of Post-Basic Education training (PBE) should be authorized to independently perform such activities as resuscitation and medication balancing while reserving invasive procedures for physicians. It is widely agreed that broadening the authority of nurses would improve the quality of NICU care even though it would increase the nursing workload.

**Conclusions:**

The study provides important input into decisions about authorizing nurses over complete practice areas rather than isolated activities; the need to remove institutional restrictions on tasks currently permitted to nurses; introducing teamwork from within the NICUs, and expanding nursing decision-making. The study reveals that there is a basis on which to to build the role of the neonatal nurse,since most NICU nurses have the suitable academic and clinical training.

## Background

Medical and technological developments, financial constraints and a shortage of physicians in the western world have raised the need to re-examine the boundaries and division of responsibility and authority between nurses and physicians, mainly with a view to expanding the role and responsibility of the nursing profession [1–3].

Israel, too, has grappled with the interface between health professions and professional boundaries (mainly of nursing). The topic is particularly important given the projected shortage of physicians, especially in medical professions identified as crisis-ridden – one of which is neonatology. The “crisis” tag stems from, among other things, the heavy workload of specialists and residents in Neonatal Units (hereafter: NICUs) in Israel’s general hospitals [4]. The number of births in Israel is steadily rising, alongside the survival of the number of newborns with a low birth weight (up to 1,500 gr), resulting in the increased numbers of hospitalized newborns [5].

According to the data of the Ministry of Health (MoH), in 2011 there were 26 neonatal wards in 27 general hospitals in Israel. The number of standard beds in these units was 624 of which 517 were for special care and 107, for intensive care. To adjust to the increase in the survival rate of underweight newborns, the units have had to increase the rate of beds for intensive care (which demands more manpower) by some 30% [5]. This figure is inconsistent with the increased number of newborns treated. One possible solution to the problem of overload might be to increase the number of approved personnel. However, the dearth of resources and shortage of neonatal physicians precludes this.

Given the growth of neonatal intensive care in Israel and the concomitant shortage of adequately trained health professionals for this setting, the aim of this study was to examine both the work processes and actual division of labor between NICU physicians and nurses, and the attitude towards nursing empowerment and expanded scope

of practice , as well as to suggest a model that suits the current working environment.

Manpower shortage, especially of physicians, introduced changes in the division of labor between nurses and physicians, affecting the distribution of responsibility and authority (based on directives of the MoH in its capacity as regulator). However, these changes have not evolved into a uniform policy that would define an accurate, differentiated division of responsibility and authority for better care.

Many countries with similar dilemmas have found alternative solutions, mostly related to broadening nursing authority and expanding the nursing role in the gray area between medical and nursing care. The most important of these is the Advanced Neonatal Nurse Practitioner (ANNP), who specializes in neonatal care in advanced studies (towards a Master's degree) and is granted wide authority [6–11]. Comparisons of the care provided by ANNPs and residents in neonatal units have found them to be of a similar quality with no significant advantage to the latter [6].

Although Israel does not yet have the position of ANNP, the MoH has recently included it in its work plan. However, over recent years, the MoH has promoted a process of broadening the authority of RNs to permit them to perform medical treatments that to date have been the exclusive purview of physicians [12]. Based on this policy, the process of Post-Basic Education (PBE) has also grown, training nurses for specific areas of practice. The courses are under the rubric of license-based training and mandatory for MoH nurses who work in special clinical practices such as NICUs. The courses provide graduates with the knowledge and authority for specific clinical interventions, much broader than those authorized for RNs with no PBE. To qualify for PBE, candidates must be RNs with at least a year’s experience and an undergraduate academic degree.

In Israel, not all nurses working in NICUs are PBE-trained due to the manpower shortage. This situation leads to the mixed training of working RNs with differential authority for specific interventions.

According to the literature review on professional boundaries, the division of labor and tasks between health professionals usually reflects such considerations as who is the most suitable service provider although it also depends on traditional roles, legislation, cultural norms and the relative balance of power between different professions. Most of the change in professional roles occurs gradually as one group assumes a new role and another relinquishes it [13, 14].

Besides the shortage of manpower, the pressure for system-wide reforms is one of the leading factors in the changing mix of skills and qualifications of the health workforce. The reforms emphasizing a restructured manpower configuration require training medical staff and even recruiting new types of manpower. Legislative changes may also enlarge or limit the boundaries while impacting the professional roles (e.g., creating new positions) [15].

Strategic planning for change should take into account the needs of professionals who are slated to relinquish certain duties. In addition, the transfer of responsibility from one profession to another requires such support as formulating evidence-based guidelines and protocols [16]. The development of this sort of plan is the first stage. In addition, the process of change should be managed systematically by adjusting the composition of staff positions, developing change methodically with staff participation, supporting staff training and development, and committing to staff assistance. It is important that change proceed in a supportive organizational culture that sees to the "soft" needs of human resources (e.g., satisfaction, personal growth and quality of care) [17].

In view of the above, this study examines in depth the actual work processes and division of labor between physicians and nurses in Israeli neonatal units, and whether there is a difference in their attitudes to broadening the authority of nurses and changing their role. It also suggests a series of activities that may help to better organize the work processes in neonatal units to make them more efficient, and to develop the role of a neonatal nursing specialist.

## Methods

The research was conducted as a case study of special-care neonatal units in Israel, and included:

a In-depth interviews with 10 physician department heads and nine head nurses of NICUs to identify and broach major problems and issues concerning the responsibility and authority of physicians and nurses. The participants of this section were recruited according to the size and location of the hospital (geography - north, center and south of Israel). Interview findings were used as a basis for constructing a questionnaire to survey the study population. Prior to presenting the questionnaire, it was reviewed by content experts, nurses and physicians. Questions included the following topics: What are the main problems in NICUs; What is the division of labor between doctors and nurses? Is there a need to change the division of authority and responsibility in the performance of nurses and doctors, and if so - why? What are the necessary changes in the division of authority and responsibility between doctors and nurses? What can be done to promote these changes?

In the in-depth interviews, the two main problems reported were a shortage of manpower, and high occupancy and over-capacity of the units. The former is due to an insufficient number of standard job slots and the difficulty of filling existing ones. All the interviewees mentioned manpower shortage as the foremost difficulty. They noted that it caused overload and staff erosion detrimental to a unit’s functioning. Interviewees hypothesized that the shortage was related to the unattractiveness of neonatology: “The lack of sufficient manpower and of approved job slots are the main problem. Part of this is due to the reluctance to deal with neonatology”; the current intensiveness of the work in NICUs compared with other departments; and an inadequate manpower standard in neonatal wards: “There is a manpower problem because the standard is for a regular department, not for an of intensive-care department.”

Another key problem raised was the inadequate physical working conditions in NICUs. These broke down into four categories: outdated and too little equipment; outdated and too few computers; low standards for equipment; high occupancy of beds detrimental to ward functioning and raising the risk of infection.

Another problem mentioned was fragmented care: The workload of neonatal physicians, including treating newborns in other departments, reduces their availability in the unit. Since nursing authority is limited, in the absence of a physician the care process is interrupted and fragmented. This generates feelings of overload and frustration, and impedes specialization in areas of care (e.g., respiratory resuscitation or hospital discharge). The situation is exacerbated by the difference in the authority of RNs and PBE graduates to intervene, and the care provided by the nurses at the two levels of responsibility to the same patients. Furthermore, in some NICUs, physicians on duty were found to belong to other departments and some had no training in neonatology. This hampers communication between physicians and nurses and possibly also the development of teamwork, complicating care and affecting its efficiency and quality.

b. The survey of physicians and nurses in NICUs relied on a self-completion, closed questionnaire administered from June 2011 to March 2012 (the full survey can be found at [18]). Twenty-two (22) NICUs from 22 general hospitals agreed to participate and were recruited for the study. The majority of the units (21) were from the public sector. The report of "Inpatient institutions and day care units in Israel, 2012" [19] categorizes hospital size by number of beds. Excluding daycare beds, there were 3 hospitals with less than 199 general beds, 4 hospitals with 200–399 beds, 5– with 400–499, and the remaining 10 hospitals had 500 beds or more. There were 432 respondents – (from 22 NICUs in the study, excluding the department heads participating in the in-depth interviews) – about 50% of the study population. Of these, there were 386 nurses (a response rate of 48%) and 46 physicians (a response rate of 46%). Forty percent of the physicians and 20% of the nurses were over 50. About half of the physicians were male, nearly all the nurses were female (97%). Most of the physicians and nurses were married (84% and 82% respectively), and most were Jewish (91% and 84% respectively). Fifty-six percent of the nurses were Israel-born compared with 43% of the physicians. Of the physicians, 80% were specialists. Of the nurses, 97% were RNs, 75% had an academic degree, and some 61% had graduated from the PBE course. The average number of years that the nurses had been in the profession was 16 vs. 20 years for the physicians. The average number of years in neonatal units was 15 for the physicians and 12 for the nurses.

### Statistical analyses

Most of the findings are expressed by categorical variables (or those measured on a nominal scale) and presented in cross-tabulation. The significance of the correlation between categorical variables was examined with both the squared Chi test and Fisher's Exact test. To compare quantitative variables of two independent categories, the t test was used to compare two groups. All the statistical tests were bi-directional and the value, p, of 5% or less, was considered statistically significant. All the statistical analyses used the SPSS program, version 18. The data from the in-depth interviews and the responses to open questions were processed using content analysis and a computer program based on Microsoft Access (2010).

## Results

### Main problems affecting NICUs

The two main NICU problems identified in the in-depth interviews were also reported by the nurses and physicians. These were the shortage of physicians and nurses (67% and 73% respectively), and the high occupancy and overcapacity (72% and 67% respectively). The lack of auxiliary and other healthcare providers was identified as another major problem (by some 42% of the respondents). Other problems were cited by a lower percentage of respondents (21% to 32%). Significantly higher percentages of nurses than physicians reported problems in the following categories: the inappropriate division of labor, physician unavailability, and the absence of known, written procedures for administrative and logistic activities (see Table 1).

**Table 1:** ^**a**^ **Major obstacles perceived by nurses and physicians in NICUs (%)**

Problems	Total N = 425	Nurses N = 380	Physicians N = 45
Shortage of physicians/nurses	72	73	67
High occupancy and over-capacity	67	67	72
Shortage of auxiliary manpower (secretary, social worker etc.)	42	42	40
Physicians’ knowledge and training	32	33	24
Shortage of research done in NICUs	32	31	37
Old equipment	31	32	22
Inappropriate division of labor between physicians and nurses*	28	29	20
Physician availability*	27	28	16
Nurses’ knowledge and training	27	28	24
Little authority of nurses	26	26	33
Absence of written procedures for administrative and logistic activities*	24	25	13
Absence of uniform regulations/guidelines for treating neonates	21	22	13

*0.05 < p < 0.1.

^a^Note: Respondents reported “to a large/very large extent” to the question: “To what extent is each of the following topics a problem in your work in the NICU?”

### Nursing activity in the NICUs

We wished to ascertain whether the nurses performed all the activities permitted them by MoH directives. Conversely, did they perform activities not permitted them by MoH, and if so, why? To the first question, 54% of the nurses and 71% of the physicians responded in the affirmative. This difference was significant (p = 0.02).

#### a. Reasons for nurses not performing MoH-Approved activities

Among respondents not performing all MoH-approved activities (Table 2), the main reason given was the absence of approval from the hospital director (60% of the nurses and 73% of the physicians). Forty percent of the nurses cited the absence of authorization from the department director, which was significantly different from the response of physicians, none of whom gave this reason (p < 0.01 – Table 2). Also, more nursing graduates of the PBE course (44%) than nurses who did not do the course (32%) cited the absence of authorization from the department director (this finding does not appear in the table).

**Table 2:** ^**a**^ **Main reasons for nurses to perform/not-perform activities (%)**

	Total N = 177	Nurses N = 166	Physicians N = 11
Reasons for non-performance of MoH-approved activities			
No approval from hospital director	60	60	73
No approval from department director***	37	40	0
Lack of knowledge/skills	18	18	18
No approval from physician on duty	1	1	0
No legal approval	5	5	0
Unknown	22	22	18
Reasons that nurses in certain situations are obliged to perform unauthorized activities			
	Total N = 177	Nurses N = 109	Physicians N = 8
Physician unavailability	40	39	50
Workload	36	37	25
Inexperienced staff	14	14	13
Other	10	10	13

***p < 0.01.

^a^Note: These totals represent respondents who do not perform all the MoH-approved activities.

#### b. Do nurses perform activities for which they have no authorization?

Fifty percent of the nurses vs. 18% of the physicians (p < 0.01) responded that in certain situations, nurses were compelled to perform activities and make decisions without authorization. Asked why, the respondents gave the main reason as the unavailability of a physician (nurses, 39%; physicians, 50%). The second most common reason was work overload (nurses, 37%; physicians, 25% – Table 2).

### Actual division of labor between nurses and physicians, and requisite nursing authorizations for NICU Work

To examine the actual division of labor between nurses and physicians, we presented interviewees with a series of activities and decisions related to NICU work. The activities were divided into two groups:

◆Activities defined by the MoH as **permitted only to PBE graduates**

◆Activities defined by the MoH as **permitted to all RNs**

We asked the interviewees about the different activities:

- Who actually performs these activities in the units?
- Are authorizations required by their units to perform these activities and, if so, which?
- Activities Defined by the MoH as Authorized for PBE Graduates

While the activities in Table 3 are permitted to PBE graduates and physicians, varying rates of PBE graduates clearly do not perform all the activities permitted them. Most PBE graduates noted that they draw blood from arterial lines (72%), and administer intravenous push medication (66%). Activities that they perform to a lesser degree are removal of arterial lines (36%), and draw blood for blood type (2%) – these activities are performed more by physicians (68% and 82%, respectively). Concomitantly, some 9% to 16% of RNs who did not do the PBE course, and are not permitted the activities listed in the table, reported that they do perform them.

**Table 3:** **Nurses and physicians reporting whether they perform specific activities** ^**a**^ **(%)**

Activity	^b^RNs N = 81	Graduates of PBE nursing course N = 235	Physicians N = 46
Arterial line blood draw	9	72	55
IV push medication	16	66	50
Upper/lower limbs or skull insertion of peripheral cannula	16	58	57
Central lines and alternate systems push medication	10	58	59
Arterial line removal	12	36	68
Draw blood for blood type and cross match	10	2	82

^a^specific activities permitted to physicians and PBE graduates only.

^b^Those not authorized to perform the activity.

We asked physicians and PBE graduates working in NICUs whether it is necessary to receive authorization (the institution, ward director or a physician) for the performance of activities permitted by the MoH.

We found that, although these activities are permitted to PBE graduates, most of the respondents believe that they do require local authorization to perform them. In general, a lower rate of nurses (3%-22%) than physicians (13%-30%) believe that there is no need for such authorization. The activities on which most PBE graduates agreed that they need authorization were: IV medication push (61%), inserting peripheral cannula (61%), drawing blood from the arterial line (58%), and IV medication push (54% – Table 4).

**Table 4:** **MoH activities permitted to PBE graduates and physicians only, and the respondents' perceived need for other necessary approval (%)**

Activity	NICU professionals (respondents)	Responses (N (PBE) =235, N (physicians) = 46)			
		No need for approval	Approval needed (from institution, director or physician)	Not performed by nurse	Unknown whether approval is needed
IV push medication	PBE nursing graduates	14	62	20	4
	Physicians	16	49	33	2
Upper/lower limbs or skull insertion of peripheral cannula	PBE nursing graduates	22	61	15	2
	Physicians	29	49	31	0
Arterial line blood draw	PBE nursing graduates	19	58	19	4
	Physicians	30	45	21	2
Arterial line removal	PBE nursing graduates	7	40	51	3
	Physicians	11	30	59	0
Central lines and alternate systems push medication	PBE nursing graduates	11	54	31	4
	Physicians	18	39	43	0
Drawing blood for blood type and cross match	PBE nursing graduates	3	35	60	2
	Physicians	13	20	2	65

b. Activities Permitted to NICU nurses

Less than 50% of RNs said that they performed the activities permitted them by the MoH (see list in Table 5). The most common of these in descending order were: connecting/disconnecting patients to and from respirators to extract secretions (49%), approval of units of blood administered by two RNs (44%), and peripheral Heparin IV flushes (31%). OTC medications are administered by only 30% of RNs.

**Table 5:** **Respondent reports of RN performance of activities permitted to all NICU nurses (%)**

	Reply: agree that nurses perform this activity		
Activity	RNs N = 81	Nursing graduates of PBE course N = 235	Physicians N = 46
Semi-automatic defibrillation	0	1	89
Connecting/disconnecting patients to and from respirators for suctioning	49	63	20
Administer unit of blood by two RNs	44	39	39
Peripheral IV Heparin flush	31	69	24
Administering OTC medication	30	17	52
Central lines and alternate systems Heparin push	14	50	24
Central line removal	9	23	70
Decision to administer adrenalin in emergency situations	0	0	91

Although the activities in Table 5 are permitted to all nurses, the staff reported that authorization was required from either the institution director or the NICU director, or that a physician was to perform them. The most salient activities dispensing with authorization, according to the nurses, were respirator connection/disconnection (65%), and peripheral Heparin IV flushes (48%). The main activities requiring authorization were approval of units of blood administered by two RNs (64%), the administration of OTC medication (58%), and the drawing of blood from central lines and alternate systems (49%). For most activities, there were no striking differences between nurses and physicians on the question of the need for authorization (thi finding does not appear in the table).

### Attitudes of NICU personnel to the staff member meant to make decisions and perform neonatal activities

We presented NICU medical staff with a list of activities and areas of decision-making, such as lumbar puncture, mechanical respiration, Full Sepsis examination, and the administration of antibiotics. We asked them which NICU professions having the proper training were meant to perform or decide on them^a^. For each item, we asked whether it should be part of the role of NICU nurses, of PBE graduates only, or of physicians only.

The data (not appearing in the table) revealed that nurses and physicians generally agreed on who should (or should not) perform some NICU activities. There was no significant difference between them. We defined agreement as “to a large extent” if at least 60% agreed on whose duty it was to perform an activity or, at most, 20% agreed that an activity was not within the purview of the professionals filling these positions.

Table 6 shows three top activities representing attitudes of NICU personnel as to who should make decisions/perform neonatal activities. There was overwhelming agreement that physicians should continue to perform their traditional activities, mainly invasive procedures such as lumbar puncture (94%) and tracheal intubation (86%), and to decide about the insertion of an arterial umbilical catheter (86%).

**Table 6:** **Three main activities by sector, that represent the attitudes of NICU personnel as to who should make decisions/perform neonatal activities**

		Reply of:			
Question asked:	Activity	RNs N = 140%	Nursing graduates of PBE course N = 228%	Physicians N = 46	Total N = 414%
Whether the task should be performed by a physician?	Performing a lumbar puncture (LP) and suprapubic aspiration (SPA)	92	95	94	94
	Neonatal intubation and mechanical ventilation	88	86	80	86
	Deciding about the need for umbilical arterial catheter insertion	84	87	85	86
Whether the task should be performed by a graduate of a PBE course?	Analyzing ventilator data and deciding on mechanical treatment plan	61	74	61	68
	Parental education regarding clinical care of the neonate at discharge	44	54	70	53
	Infection Prevention in NICUs	41	48	61	47
Whether the task should be performed by any nurse in the NICU?	Infection Prevention in NICUs	88	77	76	81
	Neonate discharge from NICUs	73	65	35	64
	Parental education regarding clinical care of the neonate at discharge	67	57	39	59

For activities to be performed by PBE nursing graduates, we found the following rates of agreement, in descending order: analysis of respiratory measures and deciding on a resuscitation plan (68%), instructing parents (53%), and prevention of infection in the unit (47%).

As to RNs who did not do the PBE course, we found widespread agreement that they were to prevent infection in the unit (81%), discharge neonates (64%) and instruct parents (59%). There was considerable agreement that they were not to perform the physicians’ traditional tasks, such as lumbar puncture, analysis of respiratory measures and deciding on a resuscitation plan, or on the need for antibiotics.

### Attitudes of NICU personnel to the division of labor between physicians and nurses, and the possibility of change

#### a. Division of labor between physicians and nurses

When asked about their division of labor, the physicians and nurses responded similarly: 61% agreed with the statement that the current division of labor permitted the unit to work efficiently, and a similar percentage (58% of the nurses, 54% of the physicians) said that it enabled quality of care. Almost half, nonetheless, expressed the belief that the division of labor should be changed (47% of the nurses, 43% of the physicians), while only a third – that it suited their existing workloads (this does not appear in table).

Table 7 shows that a similar rate of physicians and nurses responded that nurses were interested in expanding their range of activities (61% of nurses, 58% of physicians). More nurses (59%) than physicians (37%) responded that nurses wished to expand the areas of their decision-making, though 42% of the nurses believed that the physicians were interested in this as well. Some 45% of the nurses said that they were interested in expanding the range of their activities. A comparison of nurses with or without an academic degree showed that this variable is a contributing factor in the desire of nurses for broader authorities.

**Table 7:** **Respondents' attitudes to nurses' decision-making and expanded activities (%)**

	Reply: to a large/very large extent		
Category	Total N = 409	Nurses N = 366	Physicians N = 43
NICU nurses wish to expand their decision-making authority**	57	59	37
NICU physicians wish to expand the decision-making authority of nurses	42	42	39
NICU Physicians are interested in expanding the range of activities performed by nurses	61	61	58
NICU nurses are interested in expanding the range of tasks they perform	43	45	32

**p <0.01.

#### b. Expanding the areas of nursing decision-making and tasks

More than 90% of NICU physicians and nurses indicated that the main role of the physicians was clinical (consultation, diagnosis and clinical care). The rates indicating research, unit management, and controlling the care of premature infants as the role of physicians were 77%, 73%, and 73% respectively (the finding does not appear in the table).

#### c. Areas impacted by expanding the decision-making of nurses

Although most physicians and nurses believed that the expansion of areas of nursing decision-making would make nursing work more independent and efficient, a significant difference was found between them – nurses 83%; physicians 56% – p < 0.01. In addition, most of the nurses and physicians believed that it would lead to a better response in cases of intensive care (81% and 67% respectively), improve the quality of care of neonates (75% and 51% respectively), and the sense of empowerment of nurses. Most of the staff believed that expanded areas of decision-making would increase the nursing workload (76% and 58% respectively – not shown in the table).

#### d. The benefit of expanding the clinical tasks of nurses in different areas

Nurses and physicians showed similar attitudes to expanding the clinical activities of nurses though the rate of agreement with each of the statements presented was higher among the former. Most of the nurses and physicians thought that expanded clinical activities of nurses would better utilize their knowledge and skills (81%), empower them (77%), lead to more independent work (75%), offer better responses in cases of intensive care (74%), and improve the quality of care of neonates (67%). Nurses, more so than physicians, said that such expansion would also increase their workload (81% and 58%, respectively – not shown in the table).

## Discussion and conclusion

The findings point to a series of problems in NICUs related to a shortage of resources, medical manpower, and infrastructures, as well as to high occupancy and over-capacity of the units. These are consistent with the reports and findings of various committees that have dealt with the lack of manpower and insufficient beds in NICUs [20]. In addition, RNs and PBE graduates do not perform a large portion of the activities permitted them. The barriers to this are: the need for approval from higher hospital levels, mainly, the hospital director; and a lack of awareness by nurses (and also physicians) of the activities they are permitted to perform based on their clinical training. Neither physicians nor nurses know of all the NICU activities that nurses are permitted to perform. This, among other things, causes nurses to perform activities for which they are not authorized [21, 22].

Thus, the changes in professional boundaries between physicians and nurses – introduced as part of the existing NICU regulations and aimed at making procedures more efficient and solving problems identified in the study – are not executed due to the obscurity that surrounds them. Partly, it is related to the way that nursing practices are authorized – a form of slowly expanding sporadic activities not necessarily related to one another and therefore harder to keep track of. It defies the recommendations to transfer authority from one medical profession to another in an orderly fashion and according to evidence-based guidelines and protocols [16].

Like other studies in western countries [23–25], our findings indicate a high rate of support (three out of five nurses and physicians) for expanding nursing authority, and of agreement that this would improve the quality of care of premature infants even though it will increase the workload. Most physicians and nurses agree that all nurses (RNs and PBE graduates) should be authorized to independently perform resuscitation, instruct parents, handle clinical treatment and discharge neonates, prevent infection in the unit, and decide on the administration of, and removal from, phototherapy treatment. There is widespread agreement that the area of respiratory treatment can be independently performed by PBE graduates. On the other hand, there is agreement that the traditional tasks of physicians, particularly invasive procedures, should remain their exclusive responsibility. Note that another study conducted in Israel on the areas of activity of physicians and nurses found that physicians had great difficulty transferring diagnostic activities to nurses whereas the rate of consent for specialist nurses performing prescription-related activities was 45% [26].

The issues raised by the findings require that steps be taken to make NICU work more efficient.

◆ **Removing organizational restrictions from activities currently permitted to nurses** – The professional boundaries of nurses are decided by various hospital management levels. In the light of this, it is important to refresh the staff’s knowledge of the activities permitted to nurses, and to remove the administrative barriers to allow nurses to intervene as needed.

◆ **Authorizing nurses to perform complete procedures rather than sporadic tasks** – Some nursing authorizations do not apply to complete procedures, causing NICU care to be fragmented and non-continuous. This is obviously detrimental to the provision of care and prevents the nurses from building up specializations (e.g., resuscitation, discharge). It is thus recommended that the definition of NICU nursing authorizations be based on complete, coherent procedures rather than sporadic activities.

◆ **NICU-dedicated (organic) teamwork**– The multiple tasks carried out by physicians, including the care of infants and children in other departments, are a major reason for their absence from NICUs. Moreover, at some NICUs, the physicians on duty were found to belong to other departments, and some had no training in neonatology. This results in communication problems, and impedes the management and development of integrative teamwork in the interface of physicians and nurses. It is thus important that NICUs have an organic professional staff from within the ward.

◆ **Constructing a neonatal nursing specialty by expanding areas of decision-making and empowering nurses** – Among academically-trained nurses, we found a sense of efficacy and high motivation to broaden their decision-making authority. Concomitantly, we found that physicians were willing to transfer specific areas of practice to nurses. Since most NICU nurses have at least a first degree, more than half are graduates of a post-basic training course, and most have considerable experience in NICU work, this group could be developed relatively easily for the role of Advanced Neonatal Nurse Practitioner (ANNP), making NICU work more efficient and relieving the manpower shortage.

### Implications for policy

The study revealed that the following changes can be executed on the basis of existing infrastructures, work procedures, and areas of practice, which already enjoy consensus:

◆ ***Constructing clearer nursing responsibilities*** – Expanding the authority of nursing care over complete areas and at the two nursing levels (RNs and PBE graduates).

◆ ***Channeling nurses with the widest knowledge to the care of neonates posing more complex cases***: PBE graduates would be given broader authority to treat these cases, mainly the more critical NICU patients.

◆ ***Developing the position of ANNP***– Developing and implementing the role of ANNP on the basis of prior advanced studies (second degree). The role of the ANNP would include broad authority over complete areas distinct from other ward nurses and including diagnosis, decisions about medication, and advanced clinical procedures. Furthermore, ANNPs would act as a resource of knowledge for NICU nursing staff.

One of the strengths of this national study is that it is representative, covering almost all NICUs in Israel. The uniqueness of this study is that it suggests a practical model to organize NICU work processes; a program resting on an in-depth examination of actual work processes and the division of labor between physicians and nurses; and the willingness to empower nurses with greater authority. The study may well contribute to the thinking and development process concerning NICU nurses, and the definition of the role ANNP in in Israel.

## Endnote

^a^The formulation of the question was: “Given suitable training, is making decisions and performing these activities part of the role of: every NICU nurse; a PNB nursing graduate; NICU physicians?”

## Authors’ information

OT is a lecturer at the Henrietta-Szold Hadassah School of Nursing and at the School of Public Health, Hebrew University, Jerusalem, and currently focuses on Safety and Risk Management at the Hadassah Medical Organization. She holds an MSc degree in Management and a PhD degree in Nursing.

NN is a senior researcher at the Smokler Center for Health Policy Research of the Myers- JDC-Brookdale Institute. She has an MA degree in Labor Studies from Tel Aviv University.

YT is a management consultant and researcher. He is a doctoral student in public policy and government at the Hebrew University in Jerusalem, and has an MA degree in Organizational Development and Consulting.

ML is an academic consultant in the nursing administration department at Hadassah medical organization. She is a registered nurse (RN) and has a master’s degree in public health (MPH) from the Hebrew University in Jerusalem.

AT is a lecturer at the department of health systems management at the Ben Gurion university of the Negev, Beer Sheva, Israel. He is a pediatrician at the Soroka University Medical Center and has MSc degrees in both health management and biotechnology engineering.

## Abbreviations

NICU's: Neonatal Intensive Care Units
MoH: Ministry of Health
ANNP: Advanced Neonatal Nurse Practitioner
PBE: Post-basic education.
